# HIV-1 and Its Strategy for Hiding Viral cDNA from STING-Mediated Innate Immunity

**DOI:** 10.3390/ijms262311635

**Published:** 2025-12-01

**Authors:** Anna Mashkovskaia, Yulia Agapkina, Tatiana Oretskaya, Marina Gottikh, Andrey Anisenko

**Affiliations:** 1 Chemistry Department, Lomonosov Moscow State University, 119992 Moscow, Russia; 2 Belozersky Institute of Physico-Chemical Biology, Lomonosov Moscow State University, 119992 Moscow, Russia; 3 Faculty of Bioengineering and Bioinformatics, Lomonosov Moscow State University, 119992 Moscow, Russia

**Keywords:** cGAS, STING, HIV, cytosolic DNA sensor, innate immunity

## Abstract

Human immunodeficiency virus type 1 (HIV-1) is known to activate cytosolic DNA sensor pathways, such as the cGAS-STING pathway, the activation of which leads to interferon production. The primary source of this activation is reverse transcription of the viral RNA genome into cDNA, which occurs in the cytoplasm. However, the degree of cytosolic DNA sensor activation during HIV-1 infection is significantly lower compared to that induced by DNA-containing viruses or even by the related HIV-2. This can be attributed to the successful evasion of innate immune recognition pathways by HIV-1, particularly through the disruption of cGAS-STING signaling pathway activation. In this review, we summarize the available information regarding the mechanisms employed by HIV-1 to conceal viral cDNA from cytosolic DNA sensors. Deciphering these mechanisms may reveal potential vulnerabilities that could be targeted to develop novel antiviral approaches.

## 1. Introduction

The innate immune system serves to protect the host cell in response to intracellular abnormalities. This complex system is activated in response to danger-associated molecular patterns (DAMPs) or pathogen-associated molecular patterns (PAMPs), which act as a signal of cellular distress or an infection. Molecular patterns are recognized by pattern-recognition receptors (PRRs), localized in the cytoplasm or membrane-associated compartments. Once a PRR binds to a specific molecular pattern, it activates corresponding signaling pathways to induce protective responses.

**Figure 1:**
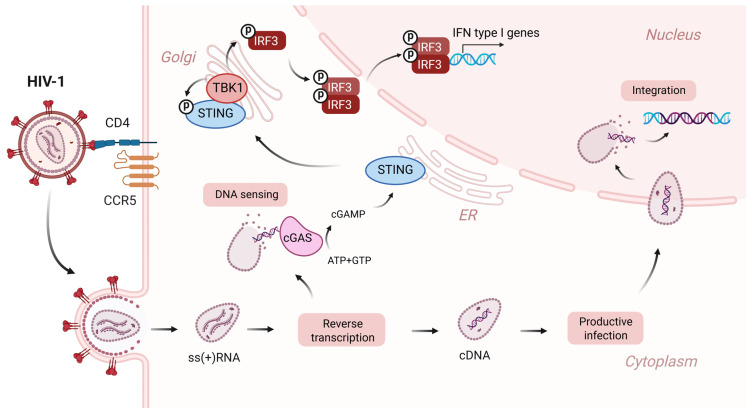
Early steps of HIV-1 replication and cGAS-STING pathway activation in response to viral cDNA. After HIV-1 capsid enters the cell cytoplasm, a cDNA copy of the viral genome is synthesized by the viral reverse transcriptase. The cDNA within the capsid is transported into the nucleus through the nuclear pore, where it becomes integrated into the host cell genome, leading to the productive infection. In the case of a premature capsid disassembly in the cytoplasm, viral cDNA is detected by cGAS, resulting in cGAMP production and activation of the cGAS-STING pathway. Created in BioRender. Rozina, A. (2025) https://BioRender.com/xs8pnzn (accessed on 28 October 2025).

The cGAS-STING signaling pathway is a cytosolic immune surveillance system that triggers type I interferon (IFN) production in response to cytosolic DNA. Cyclic GMP-AMP synthase (cGAS) has been identified as the key DNA sensor responsible for STING activation [1,2]. Upon DNA stimulation, cGAS catalyzes the synthesis of the 2′,3′-cyclic GMP-AMP (cGAMP) second messenger molecule, which is a STING agonist [1,3]. cGAMP binding promotes STING’s translocation from the endoplasmic reticulum (ER) to the Golgi complex, where STING recruits TANK-binding kinase 1 (TBK-1) [4,5]. TBK-1 in the activated STING-TBK-1 complex phosphorylates the interferon regulatory factor 3 (IRF3), causing its translocation to the nucleus and subsequent induction of type I IFN and downstream genes (Figure 1) [6,7,8]. In addition to IRF3-dependent signaling, STING can activate NF-κB signaling by contributing to IκBα (NF-κB inhibitor) degradation [4,9].

cGAS recognizes DNA in a sequence-independent manner. Therefore, activation of the cGAS-STING pathway can be observed when cells are infected with DNA-containing pathogens (e.g., bacteria or viruses), RNA viruses that utilize reverse transcription in their life cycle—including the subject of the current review, HIV-1—or even other RNA viruses if their replication disrupts the integrity of the nuclear or mitochondrial envelope, leading to host DNA leakage [10].

Retroviruses were long believed to induce weak or absent innate immune responses, except in certain cell types. One such example is plasmacytoid dendritic cells, which produce high levels of IFN in response to HIV-1 upon recognition of viral RNA by Toll-like receptor 7 (TLR7) [11]. This paradigm was challenged by the work of D. Gao et al., which opened a new avenue in HIV-1 research [12]. In this study, the authors demonstrated that HIV-1 and other retroviruses activate the cGAS-STING pathway in THP-1 cells, thereby inducing type I interferons and other pro-inflammatory cytokines. Additionally, the link between reverse transcription and DNA sensing was established [12].

Several months later, IFN production during HIV-1 infection was demonstrated in macrophages, but this process depends on another cytosolic DNA sensor—IFI16, which primarily detects hairpin regions of single-stranded DNA products of reverse transcription and, subsequently, activates the cGAS-STING pathway [13]. In the case of the main target of HIV-1—CD4+ T-cells—IFN production as a result of cGAS-STING pathway activation has also been documented, but at a lower level [14]. Despite the weaker response to HIV-1, its presence indicates an active, although impaired, immune reaction to HIV infection in these critical target cells.

Taken together, these findings clearly indicate that HIV-1 cDNA can be detected by the cGAS-STING pathway. However, the distinct behavior of the virus across different cell types indicates the presence of protective mechanisms that the virus utilizes to evade immune recognition. In this review, we will examine the primary known pathways through which HIV-1 achieves this evasion. Understanding these mechanisms is crucial for unraveling the complex interactions between the virus and the host immune system, and could provide critical insights into the origins of HIV-1’s highly epidemic nature and reveal its potential vulnerabilities.

## 2. cGAS-STING Pathway in HIV-1 Patients

The activation of the cGAS-STING pathway in response to HIV-1 has been extensively studied using various cell lines, as demonstrated above. However, considerably less data exist regarding the direct relationship between the cGAS-STING pathway and HIV-1 in patients, despite this being the most reliable indicator of whether this pathway is activated in response to HIV-1 within the human body. Before delving into the potential mechanisms by which the virus evades immune recognition, it is essential to review existing clinical information in this field. This will provide a critical basis for understanding the role of the cGAS-STING pathway in vivo during HIV-1 infection and help bridge the gap between in vitro findings and clinical observations.

First of all, elevated expression of cGAS-STING pathway components—including cGAS, STING, TBK-1, and interferon-stimulated genes (ISGs)—has been consistently observed in HIV-1 patients [15,16]. Notably, in HIV-1-infected mothers, elevated expression of STING and IFN-β genes was detected in the cord blood and decidual tissue, indicating that innate immunity can be activated in utero [17]. Some studies report that STING upregulation correlates with viremia levels [15]; others discovered the correlation between STING gene expression and the CD4+ count or CD4/CD8 ratio [18]. Interestingly, administration of antiretroviral therapy (ART) leads to the downregulation of STING pathway components, though this reduction was not accompanied by a simultaneous decrease in type I IFN levels, implying the involvement of other regulatory mechanisms for IFN expression [16,18].

Furthermore, an uncommon pattern of type I IFN induction and cGAS-STING pathway gene regulation has been observed in elite controllers (ECs) who maintain undetectable levels of HIV-1 viral load in the absence of ART [19]. Dendritic cells (DCs) from ECs, in contrast to most HIV-infected patients, demonstrate effective cGAS-dependent production of type I IFNs in response to viral reverse transcripts. Moreover, simultaneous upregulation of cGAS gene expression was observed in these cells [20]. Importantly, the enhanced intrinsic immune recognition of HIV-1 in DCs from elite controllers resulted in a stronger ability to stimulate and expand HIV-1-specific CD8+ T-cell responses [20]. This finding may at least partially explain the unusual control of HIV-1 infection observed in ECs. A recent study has demonstrated that effective IFN expression depends not only on the cGAS-STING pathway but can also rely on RIG-I-mediated sensing of viral RNA [20].

Interestingly, a different scenario is observed in some long-term non-progressors (LTNPs) who typically exhibit lower levels of viremia and a slower rate of CD4+ T-cell decline than in the general population of HIV-infected patients. In contrast to ECs, some LTNPs have an impaired STING-dependent immune response. However, only 2 of 11 studied LTNPs had homozygous single-nucleotide polymorphisms in the STING gene that disrupt interferon induction and lack of acute inflammation in response to HIV infection, while the remaining 9 patients did not have such polymorphisms [21]. Therefore, disruptions of the cGAS-STING pathway cannot be a common mechanism explaining the control of HIV infection in LTNPs.

To summarize the findings presented above, we can confidently state that the activation of the cGAS-STING pathway in response to HIV-1 infection occurs not only in model cell lines but is also observed directly in HIV-infected patients. Furthermore, in certain cases, an elevated level of activation of this pathway in dendritic cells enables effective control over the progression of HIV infection.

## 3. HIV-1 Capsid and Reverse Transcription: Balance Is the Key

The cytosolic recognition of HIV-1 cDNA relies on the accessibility of viral reverse transcripts to cytosolic DNA sensors. Consequently, the stability of the viral capsid—composed of capsid protein (CA), which protects the genetic material of the virus and facilitates reverse transcription—along with the precise regulation of its uncoating, represents a potential mechanism by which HIV-1 evades cytosolic DNA sensing.

For many years, it has been widely accepted that the uncoating of the HIV-1 capsid occurs in the cytoplasm. HIV core uncoating in the cytoplasm has been observed in different studies and was associated with reverse transcription [22,23], which facilitates the conversion of the RNA genome into more rigid double-stranded DNA, thereby increasing the internal pressure within the viral capsid. This elevated pressure contributes to capsid destabilization and subsequent uncoating [24,25]. Contrarily, inhibition of reverse transcription increases viral core stability [26].

However, the topic of where the HIV-1 capsid uncoating takes place has been a heated debate in recent years. While some studies proposed that uncoating occurs in the cytoplasm, followed by the pre-integration complex transport to the nucleus [22,23], an alternative mechanism was suggested regarding the findings of intact capsid in the nuclear pore and nucleus [27]. Early studies found that uncoating may take place at the nuclear pore [28], but according to later research, productive infection may require the capsid to enter the nucleus in an intact form and the uncoating to happen next to the integration sites [29,30]. Strikingly, nuclear pores appear to undergo structural changes to allow the conical-shaped capsid to enter the nucleus [31,32]. The capsid passage through the nuclear pore is facilitated by the selectivity of several nucleoporins towards the capsid protein, which could mean that the HIV-1 capsid is imported to the nucleus in a manner similar to transport receptors [29,33,34]. However, the documented activation of the cGAS-STING pathway in infected cells suggests that at least some viral capsids undergo disassembly in the cytoplasm, while others enter the nucleus in intact form to support productive infection.

Intact stable capsid protects the viral genome not only from cytoplasmic degradation, but also from detection by cytosolic sensors. Evidently, infection in the presence of the capsid-destabilizing small molecule PF-74 induces a more pronounced cGAS-dependent IFN response [35,36]. Infection of cells with PF74-resistant HIV particles with capsid-stabilizing mutation R143A prevents innate sensing of HIV-1 DNA [35]. In contrast, infection of THP-1 cells with HIV-1 containing a processing-defective Gag polyprotein, which causes capsid destabilization, triggers more robust cGAS-STING pathway activation compared to the wild-type virus [36,37]. The introduction of capsid-destabilizing mutations P38A or Q219A also induces higher levels of cGAS-activation, despite the concomitant reduction in cDNA production [38].

However, it is worth noting that capsid-stabilizing mutations can, in some cases, lead to an increase in the intracellular immune response. For example, introducing the E45A mutation in the capsid protein results in 20-fold higher levels of ISG expression compared to WT HIV-1. This effect, however, can be attributed to the increased formation of RT products and impaired nuclear entry of such mutant capsids, which additionally leads to elevated levels of cytosolic cDNA [38]. Overall, these findings indicate that the HIV-1 capsid serves as an effective barrier against cytosolic DNA sensors.

Notable discoveries regarding the relationship between capsid stability and cGAS-STING pathway activation were made in the study by L. Zuliani-Alvarez et al. [39]. It is well known that among 13 independent zoonoses of simian immunodeficiency viruses to humans, only one leading to HIV-1 group M has become pandemic, causing over 80 million human infections. In contrast, non-pandemic HIV-1 group O was detected in only 100,000 patients [39]. L. Zuliani-Alvarez et al. raised the question of what specific features of the M virus allow it to gain such an evolutionary advantage and spread highly efficiently through human-to-human transmission [39]. It was found that non-pandemic HIV lineages replicate less well in myeloid cells than HIV-1(M) due to the activation of cGAS- and TRIM5-mediated antiviral responses [39]. Both changes are associated with specific capsid adaptations, increasing its plasticity, as was shown by phylogenetic and X-ray crystallography structural analyses [39]. The capsid protein of HIV-1 (M) and its SIVcpzPtt parent loses the R120 residue, which forms a salt bridge with E98, increasing capsid fragility in non-pandemic HIV-1 (O). Another was the Y50Q substitution, also affecting the capsid structure. The more flexible capsid lattice in group M viruses effectively protects viral cDNA from cGAS sensing, even though inner capsid pressure increases during reverse transcription. The genetic reversal of the capsid protein in pandemic HIV-1, by introducing R120 and Q50Y substitution, restored TRIM5- and cGAS-mediated innate immune responses [39]. These findings further confirm the central role of the HIV-1 capsid in regulating the innate immune response through cGAS modulation. They also provide compelling evidence that cGAS-mediated sensing of HIV-1 is not merely a theoretical process observed only under specific laboratory conditions, but a biological phenomenon occurring in patients, and HIV-1 adapts to this reality.

In addition to internal factors affecting capsid stability, the cellular components can also modulate it. One of them is inositol hexaphosphate (IP6). IP6 is coordinated by the capsid protein amino acids R18 and K25, located in the positively charged central pore of capsomeres. The presence of IP6 in the mature capsid is critical for preventing viral core collapse during reverse transcription [40]. Since virion maturation and capsid formation occur after the particle release from a virus-producing cell and before fusion with a target cell, IP6 must be recruited into a virion from the virus-producing cell during particle assembly [41]. The incorporation of IP6 into a newly forming virion initially occurs via a distinct binding site formed by K158 and K227; however, during virion maturation and capsid assembly, IP6 relocates into the central pore [42]. Mutation K158A, impairing IP6 binding, leads to more pronounced cGAS-STING pathway activation in macrophages, but addition of T8I substitution mostly rescued IP6 incorporation and decreased cGAS activation [41].

Another factor contributing to capsid stabilization is the specific interaction of the capsid protein with host factors, such as cyclophilin A (CypA) and cleavage and polyadenylation specificity factor 6 (CPSF6). These interactions enhance viral core stability and decrease the probability of premature disassembly [30,34,43]. Collectively, both IP6 incorporation and interactions with cellular proteins enhance the ability of the viral core to protect the genome from detection by cytosolic sensors, followed by cGAS-STING pathway activation [37,41,43,44].

## 4. Host Factors Involved in HIV-1 Innate Sensing

Numerous cellular factors regulate innate immune reaction, and a number of them were shown to affect the cGAS-STING sensing of HIV-1. All known factors can be divided into two main categories: universal factors that impact the cGAS-STING pathway, and HIV-specific factors. Host factors specific to HIV infection influence the accumulation of reverse-transcription products (TREX1 and SAMHD1) or interact directly with the capsid (including CypA and CPSF6, described above, PBQP1 and NONO). Universal factors target cGAS-STING cascade activation and, subsequently, affect HIV-1 sensing (IFI16, NLRX1, and ISG15).

One of the first specific cellular factors found to negatively regulate the cGAS-STING-dependent response to HIV infection was three-prime repair exonuclease 1 (TREX1) [45,46]. TREX1 degrades cytoplasmic dsDNA and ssDNA, preventing cell-intrinsic autoimmunity [47]. DNA degradation by TREX1 prevents its recognition by cytosolic sensors, and TREX1 affects type I IFN induction in a dose-dependent manner [45,46]. TREX1 regulates the concentration of the viral cDNA during HIV infection, and can antagonize DNA sensing upstream of cGAS [13].

Another negative cGAS-STING regulator is the Sterile alpha motif and HD domain-containing protein 1 (SAMHD1). SAMHD1 restricts HIV-1 infection in certain non-dividing cells, including dendritic cells and resting CD4+ T cells. It is a phosphohydrolase, which depletes cellular nucleotide pools and, consequently, restricts viral reverse transcription [48,49]. HIV-2 and SIV, unlike HIV-1, overcome SAMHD1 restriction due to the Vpx protein, which targets SAMHD1 for ubiquitin-mediated degradation [50,51]. By limiting reverse transcription, SAMHD1 attenuates the cGAS-STING-dependent immune response; in SAMHD1-depleted cells, immune sensing of HIV-1 is much more prominent [52]. Intriguingly, some elite controllers demonstrate a higher interferon response to infection than other patients, which corresponds with reduced SAMHD1 activity [20].

Polyglutamine-binding protein 1 (PBQP1) represents an HIV-specific factor that upregulates the innate immune recognition of HIV-1. During HIV-1 infection, PQBP1 relocates next to the viral cores in the cytoplasm, where it interacts with the intact HIV-1 capsid, binding in the region of the central pore [53,54], and stimulates cGAS recruitment onto viral cDNA during capsid disassembly [55,56]. Interestingly, another cellular protein, LATS2, a component of the Hippo signaling pathway, phosphorylates PQBP1, thereby enhancing its interaction with cGAS, and, subsequently, amplifying HIV cDNA sensing [56].

Apart from cytosolic sensing, cGAS-dependent sensing of HIV DNA can occur in the nucleus as well [27]. The presence of cGAS in the nucleus has been shown, but its activation by host DNA is inhibited due to its binding to histones [27,57,58,59]. HIV DNA is protected by the viral capsid from recognition by innate immune sensors, including cGAS. Following capsid uncoating, viral DNA, especially its unintegrated form, becomes accessible. However, due to interactions with histones, this DNA adopts a chromatin-like structure that prevents cGAS activation, similar to cellular chromatin [60]. Nevertheless, cGAS-STING pathway activation through the nuclear cGAS has been observed in monocyte-derived macrophages and DCs. This process is orchestrated by the nuclear protein NONO (POU domain-less octamer-binding protein), which recognizes a conserved region in the HIV capsid and exhibits higher affinity for the HIV-2 capsid compared to HIV-1 (Kd equal to 1346 nM and 9152 nM, respectively) [27]. Upon binding to the capsid, NONO destabilizes it, recruits cGAS, and stimulates the detection of viral DNA. Although the primary focus of Lahaye et al.’s work [27] is on HIV-2 due to the higher affinity of its capsid for NONO, the data presented in the article demonstrate that cGAS-mediated sensing of nuclear viral cDNA is NONO-dependent in both HIV-2 and HIV-1 infections. Knockdown of NONO leads to decreased cDNA sensing in both cases [27]. However, these data were obtained in a specific model of cGAS-STING pathway activation, where target cells were pre-treated with the Vpx protein from HIV-2. The Vpx protein induces degradation of SAMHD1—an enzyme that hydrolyzes dNTPs. This leads to increased availability of substrates for reverse transcription and, consequently, to enhanced synthesis of cDNA. The necessity to pre-treat cells with the Vpx protein arose because, in its absence, the authors did not observe activation of the intracellular immune response to HIV-1. In the presence of Vpx, comparable levels of cGAS-STING pathway activation were observed upon infection with both HIV-1 and HIV-2, and a comparable decrease in such activation was observed upon NONO depletion [27]. Thus, data on the role of NONO in the nuclear sensing of HIV-1 cDNA require further verification. However, based on [27], such a mechanism for nuclear sensing can already be supposed. Two competing processes may occur, the fine-tuning of which determines cGAS activation. On the one hand, the interaction between NONO and the capsid facilitates capsid disassembly and recruits cGAS to the newly synthesized naked HIV-1 cDNA, leading to activation of the immune response; on the other hand, the subsequent association of cDNA with histones results in inhibition of cGAS-mediated sensing of HIV DNA.

The cGAS-STING pathway can also be modulated by general regulatory factors that are not specific to HIV infection alone. The first example is IFN-inducible protein 16 (IFI16), one of the best-studied cytosolic DNA sensors modulating the innate immune response to HIV-1 infection [13,61]. IFI16 is recruited to HIV-1 single-stranded DNA, generated during the first step of reverse transcription, through binding to its stem-rich regions [13,62]. After IFI16 activation, it helps to attract TBK1 to STING foci, promoting its activation [63]. IFI16 is shown to be crucial for sensing the cDNA products of the abortive HIV-1 infection [13,62]. Interestingly, intracellular IFI16 level correlates with the viral load and decreases after ART administration [18].

ISG15 represents another negative regulator of HIV-1 infection that modulates STING activation. During HIV-1 infection, ISG15 promotes the ISGylation of STING—a covalent attachment of ISG15 to the target protein—that facilitates STING oligomerization and enhances cGAS-STING pathway activation [64].

NLRX1 was the first member of NOD-like receptors (NLRs) found to attenuate type I IFN induction [65,66]. In the context of HIV-1 infection, NLRX1 downregulates the STING-mediated immune response by disrupting the STING-TBK1 association and preventing pathway activation [67]. NLRX1 depletion results in elevated IFN levels, which are associated with impaired nuclear import of HIV-1 cDNA. It makes NLRX1 a positive HIV-1 regulator [67].

Summarizing all the findings (Table 1), a sophisticated network of cellular factors regulates the cytosolic sensing of HIV-1. While three of the listed proteins promote cGAS-dependent sensing of HIV cDNA (PBQP1, IFI16, and ISG15), three other factors attenuate it (TREX1, SAMHD1, and NLRX1). Additionally, NONO potentiates nuclear rather than cytosolic sensing of viral cDNA via the cGAS-STING pathway. The balance between these two opposing forces ultimately determines the innate immune recognition of HIV-1.

## 5. Viral Proteins Contribute to Attenuating cGAS-STING Activation

It is clear that the capsid is only a first line of HIV-1 defense against cytosolic DNA sensors. The second one is proteins, encoded in its genome. HIV-1 encodes several regulatory proteins that have various functions, among which is cGAS-STING pathway modulation.

Vpr is a multifunctional HIV-1 protein involved in numerous processes associated with innate immunity regulation. Several studies identified Vpr as a negative regulator of cGAS-STING signaling [68,69,70]. It was revealed that Vpr localizes next to nuclear pores and disrupts IRF3 phosphorylation and translocation into the nucleus, potentially explaining its suppressive effects on this pathway [69]. Moreover, Vpr colocalizes with TBK-1 in primary DCs and dysregulates its autophosphorylation [68], which inhibits the cGAS-STING pathway. Some early studies associate viral proteins Vpr and Vif with proteasomal degradation of IRF3 factor, which should also impair innate immunity [71,72]; however, further research has failed to validate those findings [68,69]. Intriguingly, cell protein DCAF1—also known as Vpr-binding protein (VprBP)—was shown to be necessary for Vpr-mediated innate immunity inhibition. A mutant form of Vpr harboring the Q65R substitution, which impairs DCAF1 binding, failed to antagonize cytosolic DNA sensing [69]. It is well established that DCAF1 serves as the receptor component of the E3 ubiquitin ligase complex CRL4-DCAF1, which plays a crucial role in protein degradation. Vpr exploits this function: by directly binding to DCAF1, it induces the degradation of various cellular proteins [73]. Consequently, it can be hypothesized that the inhibition of innate immunity mediated by the Vpr-DCAF1 complex may be attributed to the degradation of factors involved in the cGAS-STING pathway.

It should be noted that, in addition to the inhibitory effect of Vpr on cytosolic DNA sensing, some studies have reported its stimulatory effect. However, this stimulating activity was dependent on the virus’s ability to integrate, which is somewhat surprising [14]. Such inconsistency may be explained by the difference in HIV models. For example, there is evidence that Vpr could trigger STING-mediated interferon induction independently of HIV-1 reverse transcription by activating retrotransposition of long interspersed element-1 [74]. In this case, the stable expression of Vpr after HIV-1 genome integration can lead to the activation of retrotransposition of LINE-1, which will be a main source of cDNA that triggers innate immunity pathways. Due to its involvement in multiple cellular processes, the impact of Vpr on cytosolic sensing is complex and is possibly dependent on the chosen cellular model and experimental conditions.

Two other HIV-1 accessory proteins (Vpu and Vif) have also been shown to influence cGAS-STING-mediated interferon induction. Vpu antagonizes cGAS-dependent immune sensing [14], although the effects of Vpu on DNA sensing are less pronounced compared to Vpr [70]. Interestingly, Vpu was observed to disrupt STING signaling through Nf-kB independently of cGAS signaling [14,75]. In the case of the Vif protein, it downregulates STING activity by dysregulating its posttranslational modifications by SHP-1 tyrosine phosphatase [76], and prevents TBK-1 autophosphorylation by directly binding to the kinase [68].

Two Gag-derived proteins, p17 and p6, can modulate cGAS-STING sensing of HIV-1 as well. The p6 protein inhibits cGAS-STING-mediated cDNA sensing. The E6-glutamilated form of p6 binds with the STING protein and inhibits its posttranslational K27- and K63-linked ubiquitination at K337 residue, necessary for STING-activation [77,78,79]. Surprisingly, matrix protein p17 of HIV-1 was shown to positively regulate STING signaling by inhibiting the OLA1-STING protein interaction [80]. OLA1, also known as DOC45 or GTP-binding protein 9, is a member of the Obg GTPase family. It exhibits both ATPase and GTPase activities, and regulates a broad spectrum of cellular processes, including innate immunity. OLA1 interacts with STING, preventing its translocation from the endoplasmic reticulum to the perinuclear region, and inhibiting the interaction between STING and TBK1, which is essential for subsequent signal transduction. p17 inhibits the interaction between OLA1 and STING, thereby promoting innate immune responses to cytosolic DNA [80].

Taken together, in addition to their original functions, many HIV-1 proteins can impact cytosolic sensing of HIV reverse-transcription products in addition to their main functions. Most of the involved proteins attenuate the cGAS-STING sensing, with the Vpr’s contribution remaining particularly controversial due to the complexity and diversity of its effects on the host cell. HIV-1 accessory proteins often bind directly to cGAS-STING pathway components and inhibit the posttranslational modifications and interaction with protein partners, which is critical for surpassing the signal. This strategy represents a mechanism of how HIV-1 suppresses the innate immunity and increases the chances of productive infection.

## 6. Conclusions

**Figure 2:**
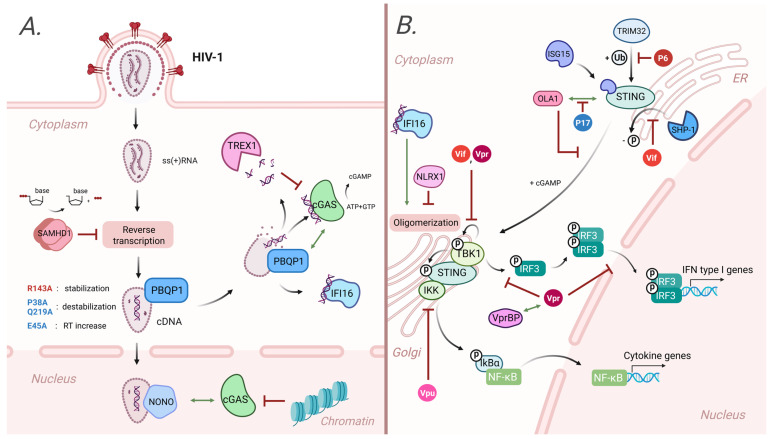
Host factors and viral proteins that influence cGAS-STING activation triggered by HIV-infection. Positive regulators are shown in blue, negative regulators are shown in red, cGAS-STING pathway components are shown in green. (**A**) After HIV capsid entry into the cell, the viral reverse transcriptase synthesizes a DNA copy of RNA genome, which can trigger cGAS. The reverse transcription is inhibited by SAMHD1, which depletes the pool of dNTPs, and as a result, inhibits innate immune activation. Capsid structure is optimized for preventing viral DNA recognition, but mutations of the capsid protein that destabilize capsid (P38A, Q219A) result in elevated innate immune activation, whereas stabilizing mutations (R143A) reduce DNA sensing. Premature capsid disassembly in cytoplasm activates cGAS-STING pathway; however, TREX1 exonuclease antagonizes this pathway by degradation of cDNA. Two capsid-binding factors recruit cGAS onto viral cDNA and upregulate innate immunity: PBQP1 promotes DNA sensing in the cytoplasm, whereas NONO binds capsid post-nuclear entry and attracts nuclear cGAS, facilitating nucleosome-free cDNA sensing right after capsid uncoating. At the same time, the sensing of cellular DNA by nuclear fraction of cGAS is inhibited by nucleosomes. (**B**) STING protein undergoes a number of posttranslational modifications crucial for cGAS-STING pathway activation. STING ISGylation (covalent attachment of ISG15 protein) is required for STING activation and oligomerization, making ISG15 a negative regulator of HIV-1 infection. Two HIV-1 proteins disrupt STING posttranslational modifications: Vif suppresses STING dephosphorylation by SHP-1 tyrosine phosphatase, and p6 antagonizes STING ubiquitination at K337 residue. Surprisingly, matrix protein p17 of HIV-1 upregulates cGAS-STING signaling by inhibiting the OLA1-STING protein interaction. After STING binds to cGAMP, it translocates from ER to Golgi complex, where STING attracts TBK-1 kinase. TBK-1 phosphorylates STING, followed by STING-TBK-1 complex oligomerization. Active TBK-1 phosphorylates IRF3 factor, which forms a dimer, translocates to the nucleus, and induces type I IFN and downstream genes. STING-TBK-1 interaction is upregulated by a cytosolic DNA sensor IFI16, and is downregulated by NLRX1 (NOD-like receptor). Two HIV-1 proteins, Vif and Vpr, antagonize TBK-1 autophosphorylation, required for TBK-1 activation. Vpr also suppresses IRF3 phosphorylation and translocation to the nucleus. Vpr’s interaction with DCAF1 (VprBP), a member of E3 ubiquitin ligase complex CRL4-DCAF1, is necessary for Vpr-mediated innate immunity inhibition. Thus, the Vpr-DCAF1-mediated inhibition of innate immunity may possibly be attributed to the degradation of factors involved in the cGAS-STING pathway. Finally, an HIV-1 protein Vpu antagonizes STING-mediated NF-κB signaling, independent of TBK-1 signaling. Taken together, the majority of HIV-1 proteins suppress STING-mediated innate immunity, when cellular factors serve to finely tune cGAS-STING pathway activation and help to recognize the HIV infection. Green arrows—positive effect on cGAS-STING pathway. Red blunt arrows—negative effect on cGAS-STING pathway. Created in BioRender. Rozina, A. (2025) https://BioRender.com/xs8pnzn (accessed on 28 October 2025).

HIV-1 was shown to be highly resistant to cGAS-STING recognition in immune cells. Although cytosolic detection of HIV-1 reverse transcripts is not abolished completely, the resulting type I interferon production is not sufficient to limit the viral progression. The evasion of immune sensing is primarily determined by the HIV-1 capsid. It serves both as a hub for the reverse transcription to take place safely, and as a “vessel” to deliver the pre-integration complex to the nucleus without cDNA detection by cytosolic sensors [39,81,82]. The capsid composition is optimal for both concealing HIV-1 cDNA and supporting the appropriate levels of reverse transcription. The fine balance between these two factors was proposed to be the driving force behind the epidemic spread of certain HIV-1 strains [38,39]. To dysregulate the cGAS-STING sensing even further, HIV-1 utilizes both host factors and the majority of its own accessory proteins, highlighting how significant it is for the virus to avoid the cytosolic detection (summarized in Figure 2 and Table 1) [14,68,69]. Notably, this evasion fails in elite controllers, leading to a robust cGAS-STING activation and extensive HIV-1 suppression [20,83].

Furthermore, in elite controllers, viral replication is controlled by increased levels and activity of innate immune cells such as DCs, NK cells, NKT cells, and macrophages. It should also be noted that, although ECs do not exhibit plasma viremia, they experience persistent HIV replication, resulting in persistent immune activation and a chronic inflammatory state. Therefore, ECs may have a higher rate of comorbidities due to the negative impact of ongoing inflammation and immune activation [84].

Based on this understanding, future therapeutic strategies may aim to specifically shift the balance in favor of immune detection. Promising avenues include the development of small molecules that affect capsid stability or blockers of capsid interactions with cellular components such as IP6, CypA, or CPSF6, such as PF74 or lenacapavir [85,86,87]. Also promising are compounds that can disturb the protective function of the capsid, prematurely exposing the viral cDNA to cytosolic sensors, or forcibly unmasking the virus from the innate immune system.

## Figures and Tables

**Table 1:** Host factors involved in cGAS-STING pathway regulation.

**Protein**	**Type**	**Effect on cGAS-STING**	**Mechanism**	**References**
TREX1	HIV-specific	Negative	Degrades cytoplasmic HIV-1 cDNA	[13,45,46,47]
SAMHD1	HIV-specific	Negative	Depletes cellular nucleotide pools and, consequently, restricts HIV-1 reverse transcription	[48,49,50,51,52]
PBQP1	HIV-specific	Positive	Interacts with the HIV-1 capsid in the cytoplasm and stimulates cGAS recruitment onto viral cDNA	[53,54,55,56]
NONO	HIV-specific	Positive	Interacts with the HIV-1 capsid in the nucleus and stimulates cGAS recruitment onto viral cDNA	[27]
IFI16	Universal	Positive	Binds to HIV-1 cDNA in the cytoplasm and, subsequently, promotes the STING-TBK1 interaction	[13,61,62,63]
ISG15	Universal	Positive	Promotes the ISGylation of STING, facilitating STING oligomerization	[64]
NLRX1	Universal	Negative	Disrupts the STING-TBK1 association	[67]

## Data Availability

No new data were created or analyzed in this study. Data sharing is not applicable to this article.
